# New light on the photocatalytic performance of NH_4_V_4_O_10_ and its composite with rGO

**DOI:** 10.1038/s41598-023-31130-9

**Published:** 2023-03-09

**Authors:** M. Nadolska, M. Szkoda, K. Trzciński, J. Ryl, A. Lewkowicz, K. Sadowska, J. Smalc-Koziorowska, M. Prześniak-Welenc

**Affiliations:** 1 Institute of Nanotechnology and Materials Engineering, and Advanced Materials Centre, Gdansk University of Technology, Narutowicza 11/12, 80-233 Gdansk, Poland; 2 Faculty of Chemistry, Gdansk University of Technology, Narutowicza St. 11/12, 80-233 Gdansk, Poland; 3 Institute of Experimental Physics, Faculty of Mathematics, Physics and Informatics, University of Gdańsk, Wita Stwosza 57, 80-308 Gdańsk, Poland; 4 Nalecz Institute of Biocybernetics and Biomedical Engineering, Polish Academy of Sciences, Ks Trojdena 4, 02-109 Warsaw, Poland; 5 Institute of High Pressure Physics, Polish Academy of Sciences, Sokołowska 29/37, 01-142 Warsaw, Poland

**Keywords:** Materials science, Materials for energy and catalysis, Nanoscale materials, Organic-inorganic nanostructures, Photocatalysis

## Abstract

Solar-driven photocatalysis has shown great potential as a sustainable wastewater treatment technology that utilizes clean solar energy for pollutant degradation. Consequently, much attention is being paid to the development of new, efficient and low-cost photocatalyst materials. In this study, we report the photocatalytic activity of NH_4_V_4_O_10_ (NVO) and its composite with rGO (NVO/rGO). Samples were synthesized via a facile one-pot hydrothermal method and successfully characterized using XRD, FTIR, Raman, XPS, XAS, TG-MS, SEM, TEM, N_2_ adsorption, PL and UV‒vis DRS. The results indicate that the obtained NVO and NVO/rGO photocatalysts exhibited efficient absorption in the visible wavelength region, a high content of V^4+^ surface species and a well-developed surface area. Such features resulted in excellent performance in methylene blue photodegradation under simulated solar light illumination. In addition, the composite of NH_4_V_4_O_10_ with rGO accelerates the photooxidation of the dye and is beneficial for photocatalyst reusability. Moreover, it was shown that the NVO/rGO composite can be successfully used not only for the photooxidation of organic pollution but also for the photoreduction of inorganic pollutants such as Cr(VI). Finally, an active species trapping experiment was conducted, and the photodegradation mechanism was discussed.

## Introduction

Population growth and rapid urbanization adversely affect the aqueous environment. Every day, industries, agriculture and households generate copious amounts of wastewater that can pollute rivers, lakes and seas. One of the major concerns is organic contaminations such as dyes or antibiotics, which are usually toxic and nonbiodegradable^1^. Even low concentrations of dyes in water systems can be very dangerous for aquatic life due to the blocking of sunlight required for photosynthesis^2,3^. Another hazardous group of pollutants is heavy metal ions, which are highly soluble in aquatic environments and nonbiodegradable and therefore tend to accumulate in living organisms either directly or through the food chain^4^. The toxic effect of many heavy metals is indisputable, and in many cases, exposure to trace amounts of these metals can cause serious damage to human health and the ecosystem^5^. For example, Cr(VI) is highly carcinogenic. U.S. Environmental Protection Agency listed it in the top 20 on the Hazardous Substance Priority List^6^, while the recommended WHO safe limit of Cr(VI) in drinking water equals 0.1 ppm^7^. Unfortunately, industrial wastewater coming from the manufacturing of paints, papers, preservatives or metal surface treatment (electroplating) can still be listed as the main source of Cr(VI). Hence, it is very important to remove the abovementioned pollutants from wastewater and protect the environment. Many methods of treating wastewater have been proposed, such as adsorption, filtration, coagulation, or photocatalytic degradation^8^. In particular, the latter is highly promising since it not only removes contaminations but also leads to their decomposition. Thus, compared to other popular methods in which contaminants are transferred from one phase to another, photocatalysis wins because of the absence of secondary pollution. Moreover, the process is usually fast, often uses a natural source of light and can be carried out under ambient conditions. Due to the above advantages, searching and developing new photocatalytic materials is an important ongoing research topic^9,10^. Among the various materials, metal oxides have been extensively investigated for the photodegradation of water pollutants^11,12^. Vanadium-based oxides are particularly promising because of their efficient visible light-harvesting ability (owing to their narrow band gap, E_g_ ~2eV), high chemical stability and significant catalytic activity^13,14^. Many metal vanadates have been proposed as promising solar-driven photocatalysts. Examples include Cu_3_V_2_O_8_^15^, Ag_3_VO_4_/AgVO_3_^16^, InVO_4_^17^ or BiVO_4_^18^, which are the most well-known catalysts in this field. Recently, we proposed using a simple potassium salt (potassium formate) as a promising alternative for the synthesis of visible-light-driven photocatalysts. The obtained potassium vanadates (KV_3_O_8_, K_2_V_6_O_16_•nH_2_O) exhibited excellent photocatalytic activity, resulting in more than 90% degradation of methylene blue (MB) within the first 30 min^19^.

In this study, we present an efficient NH_4_V_4_O_10_ photocatalyst that is composed of NH_4_^+^ instead of metallic cations and can be synthesized via a facile hydrothermal method from an easily accessible NH_4_VO_3_ precursor. Moreover, we propose to combine it with rGO and investigate for the first time the use of such a composite for the photodegradation of water pollutants. The idea of the combination of vanadates with rGO to enhance the photocatalytic properties is known from the literature. It was already shown that the transfer of excited electrons from vanadate to rGO is possible in such composites^20^. This phenomenon can positively affect e-/h + separation and inhibit the adverse recombination process. Usually, the preparation of vanadate/rGO composites requires two steps: separate synthesis of vanadates and their further reaction with carbonaceous materials. A huge advantage of the proposed NH_4_V_4_O_10_/rGO composite is its one-pot synthesis, involving simultaneous hydrothermal reduction of NH_4_VO_3_ and GO. Notably, the NH_4_V_4_O_10_/rGO composite was already reported and proposed as an efficient electrode material for capacitive deionization^21^ and aqueous zinc-ion batteries^22,23^. However, it has never been tested in photocatalytic applications.

The photocatalytic activity of synthesized NH_4_V_4_O_10_ and NH_4_V_4_O_10_/rGO was evaluated towards the oxidation of methylene blue and the reduction of Cr(VI) under simulated solar light illumination. The excellent performance in MB photodegradation is attributed to the high content of V^4+^ surface species (which promotes charge carrier separation) as well as the developed surface area (which ensures more active sites for the adsorption and photocatalysis process). Moreover, we show that the combination of NH_4_V_4_O_10_ with rGO widens its application, and the proposed composite can be used not only for photooxidation of organic pollution but also for photoreduction of inorganic pollutants with Cr(VI) as an example. The addition of rGO also enhances light absorption in the visible region, accelerates the photooxidation reaction, and ensures cyclic stability.

## Results and discussion

### Structure characterization

**Figure 1:**
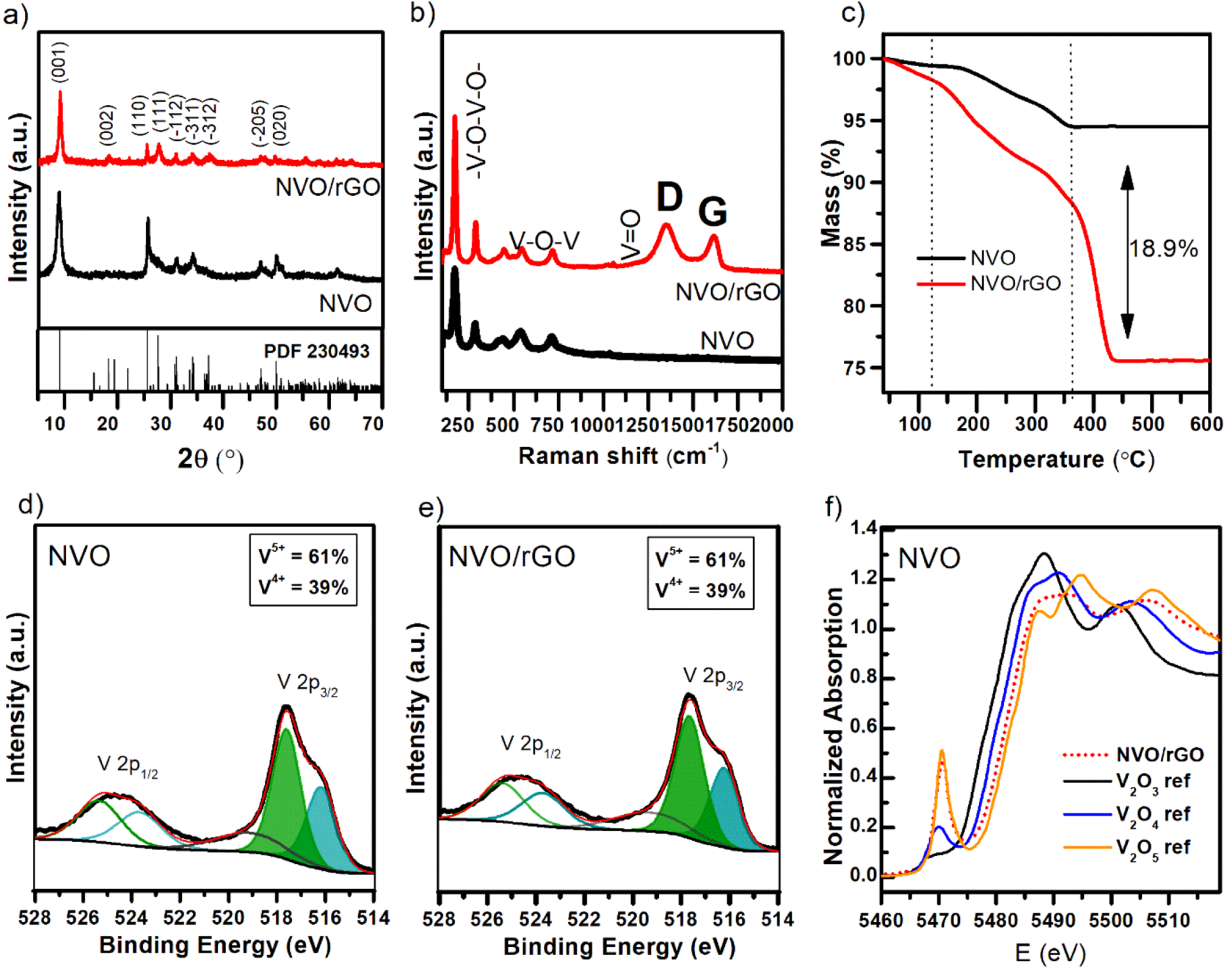
Structural analysis of NVO and NVO/rGO: (**a**) XRD, (**b**) Raman, (**c**) TG (air, 10 °C/min), (**d**,**e**) V 2p XPS spectra and (**f**) V K-edge XANES spectra (the inset shows the relation between the vanadium oxidation state and the edge position).

The XRD patterns of NVO and NVO/rGO are presented in Fig. 1a. All diffraction reflexes correspond well with the ammonium vanadate phase NH_4_V_4_O_10_, PDF no. 230493 (ICSD), which corresponds to a monoclinic structure with lattice parameter values of a = 11.57 Å, b = 3.65 Å, and c = 9.81 Å)^24,25^, which is characterized by a layered structure built from corner-shared VO_6_ layers and NH_4_^+^ cations between them. The strongest line corresponds to the 001 plane, which indicates preferential growth along the c-axis. The absence of any other reflexes indicates that a pure phase was obtained for both samples. In addition, characteristic diffraction reflexes of rGO were not observed for the NVO/rGO composite due to the intrinsic weak diffraction intensities of the graphene phase. Moreover, it has also been reported that no rGO reflexes are observed when carbon material is homogeneously dispersed in the composite^21,26^. Broad reflexes for both samples indicate a small size of ammonium vanadate crystals, which is in agreement with further SEM observations. Raman spectroscopy analysis was performed to identify the characteristic bonds of NH_4_V_4_O_10_ as well as to confirm the presence of rGO in the composite (Fig. 1b). The bands observed in the low wavenumber region (< 1000 cm^-1^) are associated with V–O bonds and are similar to those reported for NH_4_V_4_O_10_^27^. The band at 147 cm^-1^ is due to the layered structure of NH_4_V_4_O_10_ and bending vibration of (V_2_O_2_)_n_ chains^28^. The band at 285 cm^-1^ originates from the bending vibration of O-V = O^28^. The symmetric and antisymmetric stretching modes of V–O-V appear at 540 cm^-1^ and 720 cm^-1^, respectively, while the bending mode appears at 450 cm^-1^^29^. In the NVO/rGO spectrum, two new distinct modes located at 1351 cm^-1^ and 1616 cm^-1^ are the D and G bands, which are characteristic of graphene-based materials and prove the presence of rGO. The G band arises from the in-planar stretching of symmetric sp^2^ C–C bonds in graphite-derived carbon materials. The D band is attributed to the presence of interruptions that occurred in the symmetric hexagonal graphitic lattice, such as heptagon and pentagon rings, edge defects, and functional groups. Such disruptions are typical for rGO and are beneficial for composite preparation, as the folding of the defective graphene sheets facilitates the homogeneous distribution of the material in the final structure. The characteristic bonds of NH_4_V_4_O_10_ and rGO were further confirmed by FTIR analysis (Fig. S1). The spectrum of pristine NVO contains a few pronounced bands associated with the vibrations of V–O bonds: symmetric and asymmetric stretching of V–O-V (535 cm^-1^ and 740 cm^-1^) and symmetric stretching of V = O (980 cm^-1^)^30^. The observed splitting of the V = O band indicates the existence of V^5+^ and V^4+^ in the structure and is typical for NH_4_V_4_O_10_, which belongs to the mixed-valence vanadium compounds^28^. Absorption bands at 1405 cm^-1^ and 3180 cm^-1^ correspond to the bending and stretching modes of N–H in NH_4_^+^, respectively^31^. Bands located at 1630 cm^-1^ and 3450 cm^-1^ can be assigned to the O–H bending and stretching vibrations of adsorbed water. In the NVO/rGO spectrum, a new band located at 1560 cm^-1^ can be clearly observed, which corresponds to the C = C skeleton vibration of the graphene sheet ^32^. In addition, weak bands associated with carbon‒oxygen bonds can also be observed for NVO/rGO. This implies that after the hydrothermal reaction, some oxygen functionalities are still present on the surface of the graphene sheets. Such residual groups are a typical feature of rGO and are beneficial for attracting dye molecules^33^ and metal ions.

The content of various components within the vanadate/rGO composites can be estimated by TGA under an air atmosphere. Calculations are based on the difference in mass loss between pure ammonium vanadate and its composites with rGO^22,34^. As shown in Fig. 1c, the total mass loss for pure NVO up to 600 °C is 5.5%, and two main regions can be distinguished, which correspond to the removal of adsorbed water (before 110 °C) and ammonium groups (110–370 °C) by the release of ammonia and water. The 4.9% loss in the second region indicates the absence of crystal water, and its value agreed well with the molar mass of the ammonium group in the nonhydrated NH_4_V_4_O_10_ phase. Compared to NVO, NVO/rGO reveals an additional sharp mass loss (12.9%) with a maximum ca. 410 °C, which is attributed to the combustion of rGO sheets in the composite ^22^. Moreover, the mass loss in the range of 110–370 °C is doubled. This is caused by the decomposition of residual oxygen-containing groups of rGO. Considering the differences in the total mass loss between both samples, the content of rGO in the NVO/rGO composite was calculated to be equal to 18.9%. To investigate the surface oxidation state of V in the studied samples, XPS analysis was carried out. The high-resolution XPS spectra of the V 2p region (Fig. 1d,e) reveal two peaks located at 524.5 eV and 517.3 eV assigned to the V 2p_1/2_ and V 2p_3/2_ doublets. The observed asymmetry of the peaks indicates the coexistence of V^5+^ and V^4+^ and confirms the mixed-valence nature of the obtained samples. Thus, for further analysis, V 2p_3/2_ was deconvoluted for two peaks at 517.6 eV and 516.2 eV corresponding to V^5+^ and V^4+^, respectively. According to the fitted peak areas, the ratio of V^5+^/V^4+^ in both samples is similar and equal to 1.6, which is almost two times lower than the theoretical value for NH_4_V_4_O_10_. Taking into account the stoichiometry of NH_4_V_4_O_10_, the theoretical content of V^5+^ is 75%, while that of V^4+^ is 25%. At the same time, it should be kept in mind that XPS is a surface-sensitive method, and the obtained results indicate a high content of V^4+^ (39%) on the surface, which may be different in the bulk. Furthermore, the valence state of vanadium in the bulk determined from XAS analysis was 4.75. As shown in Fig. 1f, the edge position for NVO/rGO lies between the energies of the V_2_O_5_ and V_2_O references. It should be noted that transmission mode XAS analysis refers to the bulk oxidation state, while XPS is a surface-sensitive method. It is known from the literature that V^4+^ species can be generated on the surface of vanadates during their synthesis^35,36^. It was also proven that the presence of surface V^4+^ species is also beneficial for the photocatalytic process and will be discussed later.

**Figure 2:**
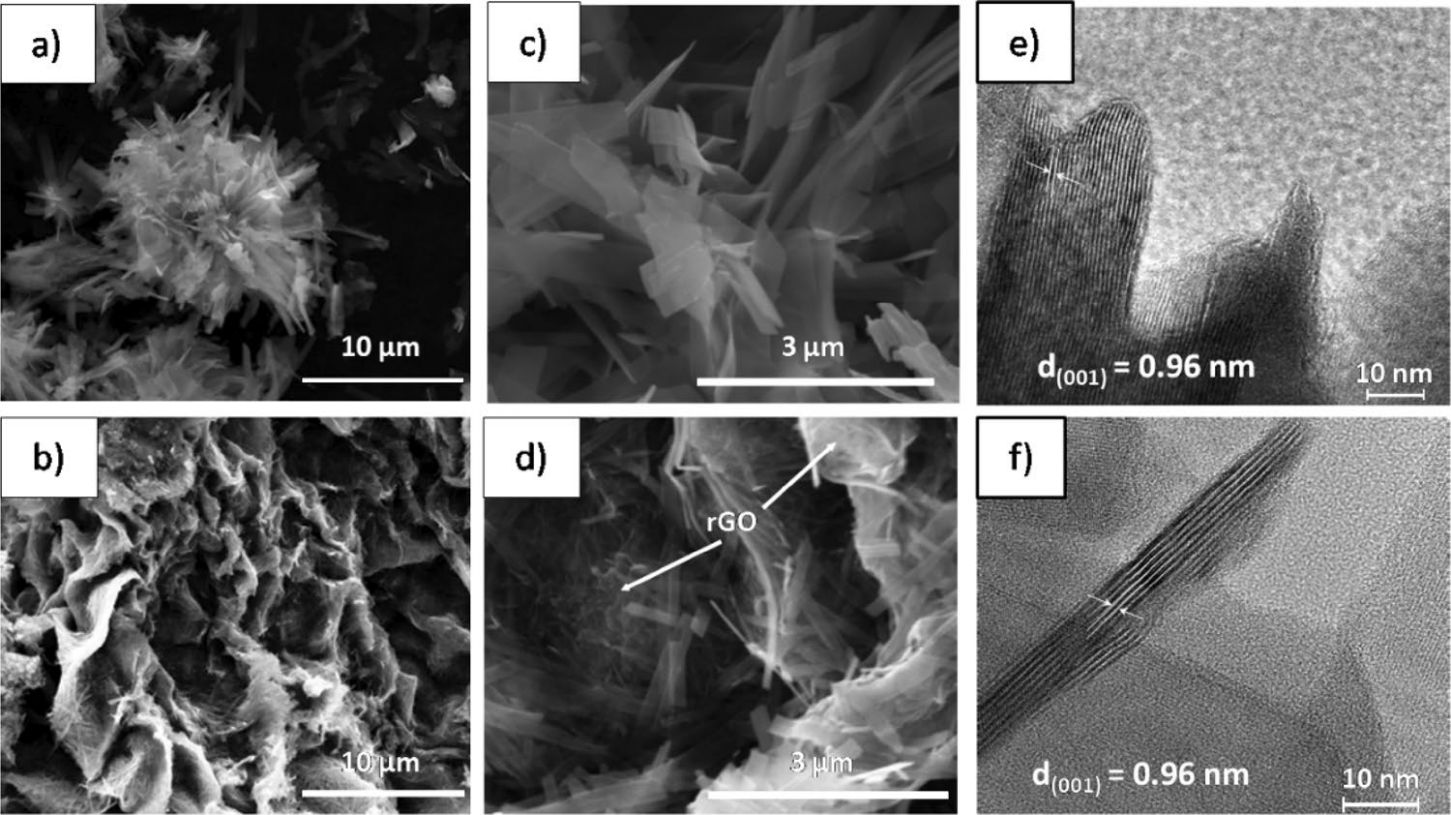
SEM images of NVO (**a**,**c**) and NVO/rGO (**b**,**d**) and (**e**,**f**) high-resolution TEM images of NVO and NVO/rGO, respectively.

The morphology of the prepared samples was investigated using SEM. As shown in Fig. 2a,c), NVO exhibits a flower-like microstructure with an average diameter size equal to 10 µm. The observed structures are built of nanobelts with a length of 2–7 µm and a width of 500 nm. The thickness of the nanobelt is approximately 40 nm. By comparison, the NVO/rGO sample possesses a very different morphology. As seen in Fig. 2b,d), the composite is characterized by a porous structure made of connected nanobelts, which are wrapped with rGO sheets. The width of the nanobelts is smaller than that for NVO and equal to 200 nm, which can be attributed to the slower growth rate limited by rGO. Such a unique structure should facilitate the migration of aqueous solution, ensuring better contact of pollutants with active sites when used as a photocatalyst material for water treatment. It is also expected that the hierarchically organized structure of the NVO/rGO composite ensures its stability, which will improve the reusability of photocatalysts. Figure S2 shows the N_2_ adsorption–desorption isotherms of NVO and NVO/rGO. Both samples exhibit a type-IV isotherm with a hysteresis loop typical for mesoporous materials. It is known that the existence of mesopores in the photocatalyst facilitates the accessibility of pollutant molecules, enhancing its effectiveness^37^. The pore size distribution, calculated with the BJH model, reveals that for both samples, the pore radius is in the range of 2–40 nm. NVO possesses mostly small pores with a mean radius size equal to 2 nm, while NVO/rGO is characterized by a bimodal size distribution with pore radii centered at 2 nm and 8 nm. The cumulative pore volume of NVO/rGO (0.19 cm^3^/g) was more than two times larger than that of NVO (0.08 cm^3^/g). In addition, the specific surface area was determined to be 28 m^2^/g and 33 m^2^/g for NVO and NVO/rGO, respectively. As expected, the composite shows a larger surface area, owing to the high intrinsic surface area of rGO and the self-organized porous structure of NVO/rGO. The high-resolution TEM images of NVO (Fig. 2e) and NVO/rGO (Fig. 2f) show that the lattice plane is 0.96 nm and corresponds to the characteristic (001) plane of NH_4_V_4_O_10_, as observed from the XRD pattern (shown in Fig. 1a).

**Figure 3:**
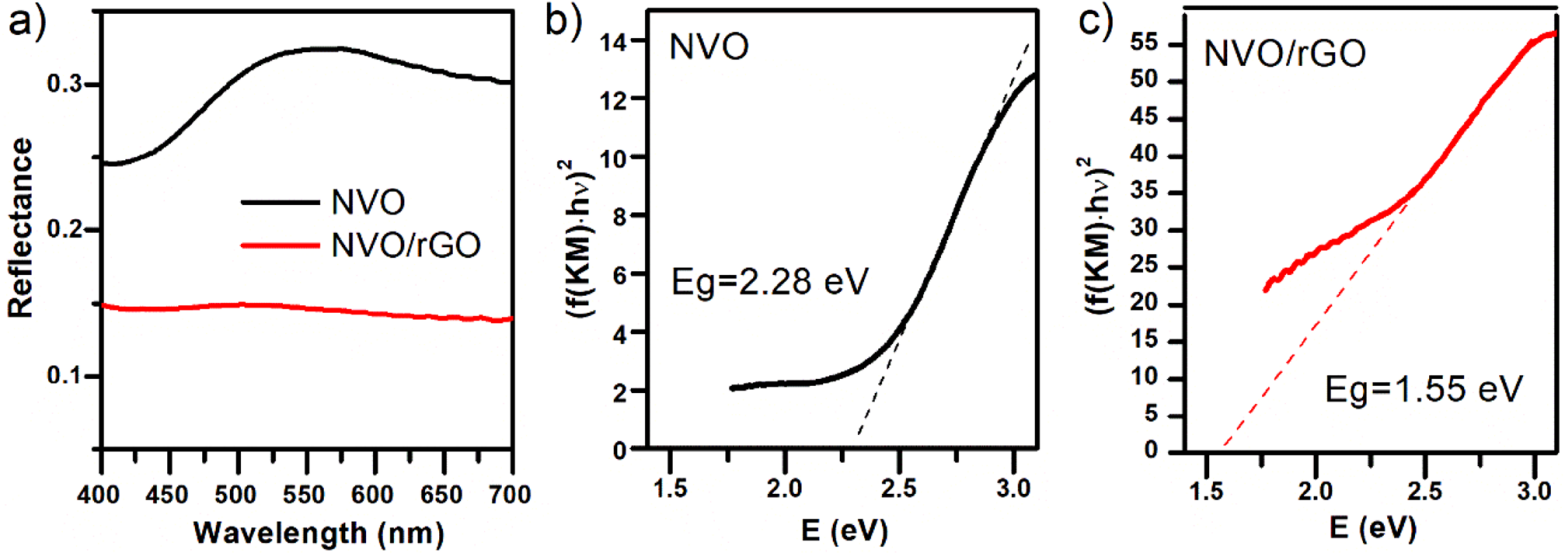
Optical properties of NVO and NVO/rGO: Reflectance spectra (**a**) and the (f(KM)·hν)^2^ vs hν plots resulting from the UV‒Vis reflectance spectra with corresponding energy band gaps for NVO (**b**) and NVO/rGO (**c**).

Diffuse reflectance UV‒Vis spectroscopy was applied to study the optical properties of both materials. The band gap energies were calculated using the Kubelka–Munk method based on the diffuse reflectance spectra presented in Fig. 3a. The Kubelka–Munk model is the basis for determining the band gap of thick, optically rough powder samples. Following^38^, (f(KM)∙hν)^1/n^ (for n = 1/2 assuming a direct bandgap) was plotted as a function of the incident photon energy (hν), and the results are depicted in Fig. 3b,c for NVO and NVO/rGO, respectively. The band gap of both materials was determined by extrapolation of the linear region of (f(KM)∙hv)^2^ vs hv to y = 0. The intersection point of the extrapolated line with the abscissa gives the value of E_g_. As shown in Fig. 3b, the obtained energy band gap for NVO is 2.28 eV, which is in good agreement with the previously published value^39,40^. The optical band gap of NVO/rGO was determined to be 1.5 eV. The reported values for rGO cover a wide range between 0.02 and ca. 2 eV^41–43^, but the E_g_ of rGO is generally lower than that of NVO; thus, the bandgap narrowing in a synthesized composite is not surprising. The band gap decrease upon adding rGO to known photocatalysts, such as ZnO or TiO_2,_ was also reported in the literature^44,45^. The positive influence of rGO on photocatalytic activity has been recently reviewed by Mondal et al.^46^. The results obtained by us are in line with the worldwide trends summarized in this review.

**Figure 4:**
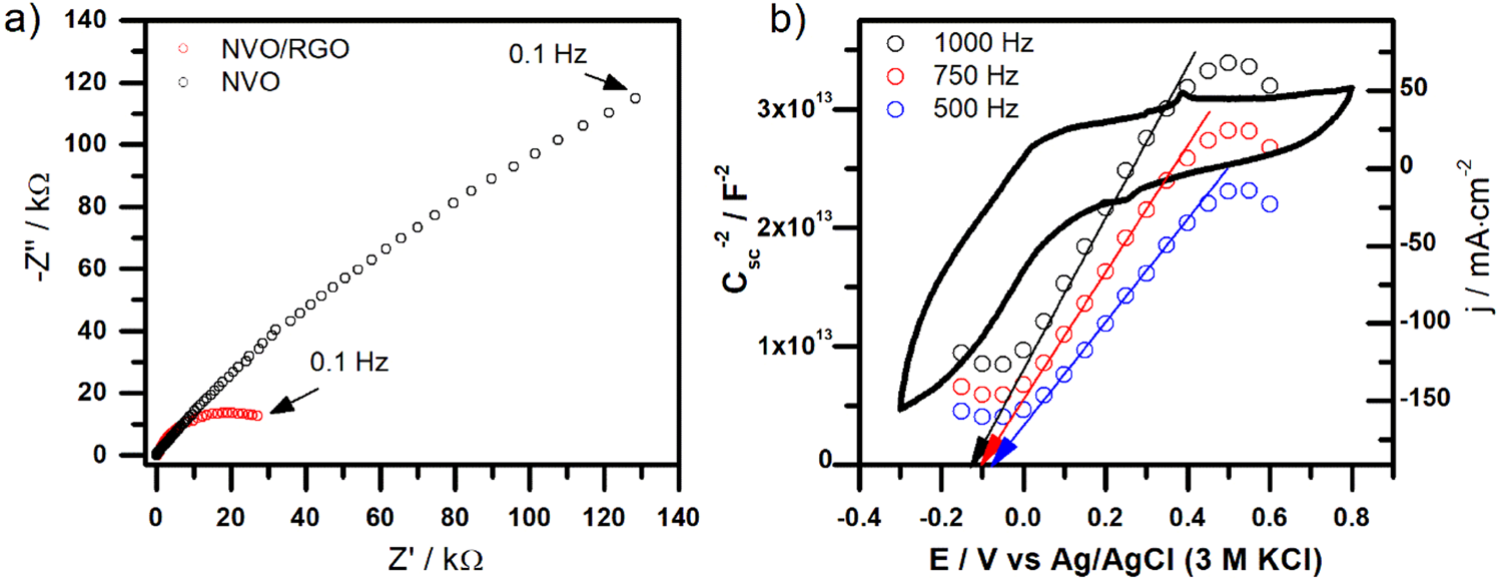
(**a**) Typical Nyquist plots recorded for NVO/RGO and NVO at rest potential equals to about 0.3 V and (**b**) the cyclic voltammetry curve of NVO, recorded in 0.2 M K_2_SO_4_, scan rate: 50 mV/s and Mott–Schottky plot of NVO electrode at different frequencies.

The photoemission spectra of the samples were measured using UV radiation as the excitation source. The results are shown in Figure S3. The intensity of emission is very low for both NVO and NVO/rGO. Nevertheless, a band at approximately 460 nm was registered. Similar behaviour was already observed for K-containing vanadate—K_2_V_6_O_16_·0.65H_2_O^19^. Taking into account that the energy band gap estimated from the UV‒vis spectrum equals approximately 2.28 eV (~ 543 nm), the observed emission band is not related to the conduction to valence band transition. The emission band with a maximum at 460 nm was reported for V_2_O_5_ nanostructures and was associated with recombination of electrons photoexcited to higher than conduction band edge levels^47^. Here, despite the very low intensity of emission, it can be seen that the presence of rGO in the composite quenches the photoluminescence significantly. It is often an indication that recombination is hindered due to the transfer of photoexcited electrons to rGO, and the separation of charge carriers that can participate in photocatalytic processes is improved. The comparison of EIS spectra recorded at rest potential is shown in Fig. 4a. As expected, the presence of highly conductive rGO affects the electrical properties of the material. The resistance of charge transfer, seen as the diameter of the semicircle on the spectra, is evidently diminished, suggesting that the conductivity of the sample is enhanced due to the presence of rGO. To compare the electrochemical activity of both materials using the cyclic voltammetry technique, see Figure S4. In the case of bare NVO, reversible redox activity, probably associated with the electrochemical activity of surface V-containing groups, was registered. Additionally, the cathodic current can be seen at a potential lower than approximately 0 V vs Ag/AgCl (3 M KCl), which can be related to the conduction band level. Such a shape is characteristic of n-type semiconductors because n-type semiconductors can act as cathodes for both illuminated and dark conditions^48^. The CV curve of NVO/rGO is much more complex and contains 4 electrochemical activities in the measured potential range. Additionally, the measured current is much higher than that for NVO. Despite the fact that both materials differ from each other by the presence of rGO, the additional electroactivities do not originate from the rGO^49^. Their presence is related to the better electrical properties of the investigated electrode material and facilitated electrochemical activity of NVO. Mott-Schottky analysis was performed to estimate the flat-band potential of NVO (see Fig. 4b). The slope of the 1/C_sc_^2^ is positive, confirming that NVO is n-type semiconductor. Some frequency dispersion was observed; however, it can be concluded that the flat-band potential is approximately -0.1 V vs Ag/AgCl (3 M KCl) (0.52 V vs NHE). Notably, the estimated value is close to the maximum of the cathodic peak seen on the CV curve, which is consistent and characteristic with the n-type conductivity (the conduction band edge and flat-band potential are close to each other).

### Photocatalysis of organic pollutants

**Figure 5:**
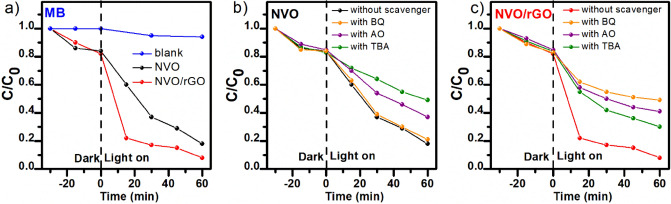
Photocatalytic degradation of MB under simulated solar light: (**a**) C/C_0_ vs t plot and (**b**,**c**) active species trapping experiments with the addition of BQ, AO, and TBA as scavengers for O2- radicals, holes h + , and hydroxyl radicals ·OH, respectively.

The photocatalytic activity of the prepared samples was first evaluated by the degradation of the organic dye methylene blue (MB) under simulated sunlight. Before the photocatalysis experiments, the adsorption of MB on the surface of the tested powders was evaluated under dark conditions (Figure S5a). Adsorption–desorption equilibrium was achieved after ca. 30 min for both samples, and approximately 20% of the dye was adsorbed. The observed good adsorption ability comes from the high specific surface area of the tested materials (confirmed by BET) and is essential for the photocatalysis process, ensuring improved contact between the pollutant molecules and reaction active sites^50,51^. Figure 5 presents the results of the photocatalytic performance of NVO and NVO/rGO toward MB degradation. An illuminated MB solution without the addition of photocatalysts (blank) was also added for comparison and revealed a negligible photolysis effect. Similarly, the photocatalytic performance of pure rGO was measured as a control experiment and is presented in Figure S5b. After 60 min of illumination, NVO degraded 82% of MB. To the best of our knowledge, only three publications devoted to the usage of NH_4_V_4_O_10_ in photocatalytic applications have been reported. For example, the experiments conducted by Aboood et al. reveal that the cross-like NH_4_V_4_O_10_ nanobelt arrays do not show any catalytic action for the degradation of rhodamine B after 210 min under visible light^52^. However, scientists have shown that photoactivity can be induced by calcination (above 400 °C) of the as-obtained structures and their transformation to V_2_O_5_. Two other works are devoted to the heterojunction of NH_4_V_4_O_10_ with quantum dots (CdS and C-dots). Y. Zhang et al. designed an efficient ternary C-dots/SrTiO_3_/NH_4_V_4_O_10_ catalyst for the removal of sulfamethoxazole, aureomycin hydrochloride, and ciprofloxacin residues in aqueous samples^26^. The reported photocatalytic degradation efficiency of the antibiotics for pure NH_4_V_4_O_10_ was lower than 20%, which was 4–5 times lower than that for the heterojunction. S. Le prepared an intercalated CdS/NH_4_V_4_O_10_ composite that degraded 90% of amoxicillin within 120 min under simulated sunlight, while pure NH_4_V_4_O_10_ can degrade 50%. Taking into consideration the above examples, the photocatalytic efficiency obtained in this work for pure NH_4_V_4_O_10_ is unexpectedly high. It is suggested that the excellent photoactivity of NVO results from the high content of V^4+^ on the surface and well-developed surface area. It has been demonstrated that V^4+^ can trap photogenerated electrons and promote efficient charge separation, enhancing photocatalytic performance in water splitting and degradation of pollutants^35,53,54^. It has also been reported that the existence of V^4+^ can lead to the formation of intermediate defect energy states and the widening of the optical absorption range^55^. V^4+^ species can be introduced into the structure of various vanadates in several different ways, such as doping with other elements^54^, post-treatment (e.g., calcination^56^, plasma modification^53^) or hydrothermal reduction with reducing additives^35^. The last approach was also used in this study, and oxalic acid was added during the synthesis. As confirmed by physicochemical characterization, oxalic acid serves as a reductant and induces the formation of V^4+^ on the surface of NH_4_V_4_O_10_ during the hydrothermal process. It should also be noted that oxalic acid can act as a forming agent during the hydrothermal synthesis of ammonium vanadates^22^. Thus, it is suggested that its addition led to the formation of 3D flower-like microstructures, as observed here for bare NH_4_V_4_O_10_.

Moreover, herein, we propose to combine NH_4_V_4_O_10_ with rGO. This can be done during a hydrothermal reaction, which in contrast to the synthesis of previously mentioned heterojunctions is a one-step process. Another advantage of such synthesis is a hierarchal porous structure that is formed during the hydrothermal reaction. In contrast to the NVO sample, in the case of the NVO/rGO composite, the morphology is probably affected by both oxalic acid and graphene oxide. As shown in Fig. 5, the coupling of NH_4_V_4_O_10_ with rGO resulted in improved photocatalytic performance, which reached 92% within 60 min.

**Figure 6:**
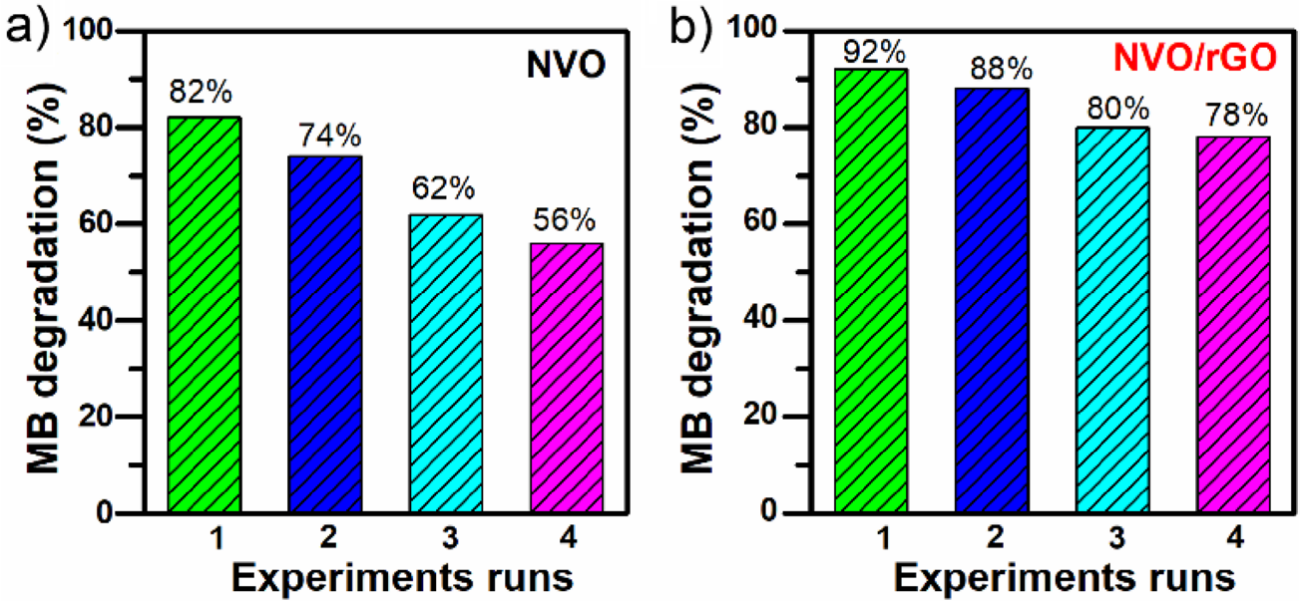
Reusability of the NVO (**a**) and NVO/rGO (**b**) photocatalysts for the degradation of MB.

The kinetics of MB photodegradation were further studied by plotting -ln(C/C_0_) as a function of irradiation time (Figure S6). In the case of NVO, the is most likely pseudo-first-order kinetics according to the Langmuir–Hinshelwood model, and the calculated rate constant k was equal to 0.0251 min^-1^. For NVO/rGO, the mechanism of MB photodegradation can be more complex (as indicated by the low linear regression coefficient); however, it can be clearly seen that at the beginning of illumination, the process is much faster. To identify the main reactive species involved in the photodegradation process, further tests were performed in the presence of different scavengers. Benzoquinone (BQ), ammonium oxalate (AO), and tert-butyl alcohol (TBA) were used as scavengers for ·O2- radicals, holes h + , and hydroxyl radicals ·OH, respectively. As depicted in Fig. 5b-c), compared with the photocatalytic process without scavengers, the removal rate of MB by NVO decreased by 47% and 29% in the presence of TBA and AO, while no change was observed with the addition of BQ. This indicates that ·OH and h + play a critical role in MB photodegradation. In contrast, the degradation of MB with the composite is highly suppressed by BQ, indicating the main role of ·O2- radicals in the process. The addition of AO and TBA also shows a significant inhibitory effect, which indicates that h + and ·OH also contribute to the degradation of MB. The reusability of NVO and NVO/rGO was further investigated by performing 4 consecutive photodegradation cycles for MB (Fig. 6). The results revealed that the activity of NVO gradually decreases, and in the 4^th^ cycle, the efficiency drops to 56%. Better stability was observed in the case of the composite. The results obtained for NVO/rGO show that the degradation efficiency was maintained at a good level during each reaction cycle during irradiation, and consequently, the photocatalyst could be reused.

### Photoreduction of inorganic pollutants

**Figure 7:**
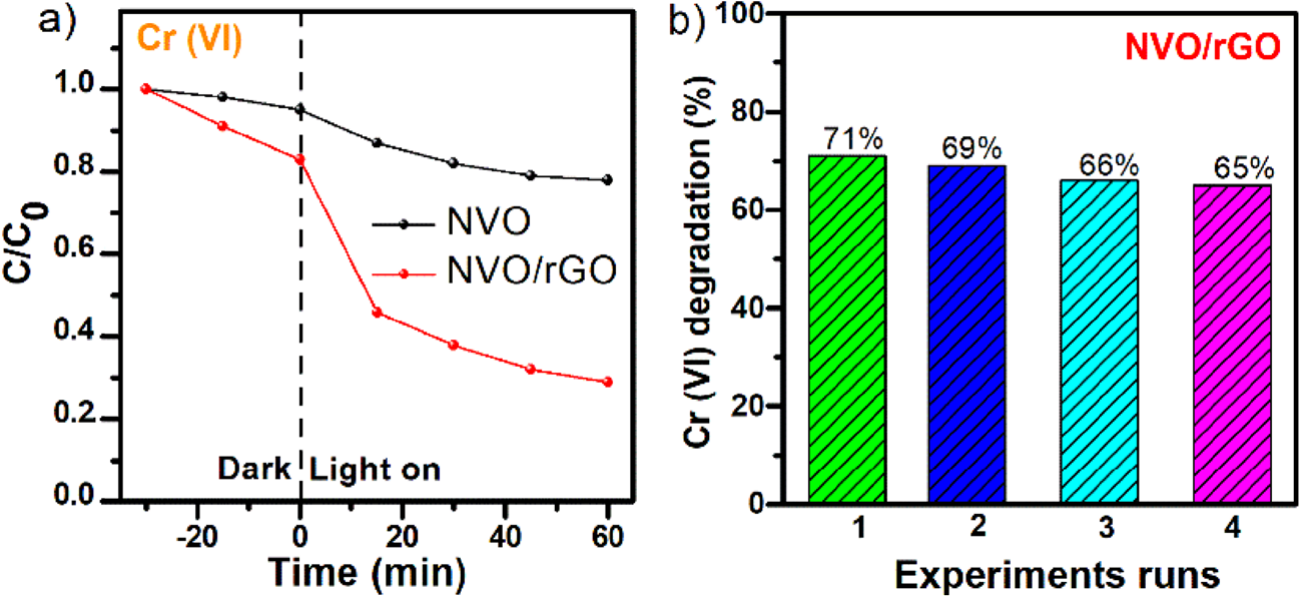
The photocatalytic reduction of Cr(VI) to Cr(III) under simulated solar light: (**a**) C/C0 vs t plot and (**b**) reusability of NVO/rGO.

The obtained photocatalysts were also used in the Cr(VI) photoreduction process at pH 3. It is well known that under acidic conditions, Cr(VI) is mainly present in the form of HCrO_4_^−^ or Cr_2_O_7_^2−^, which eventually promotes the reduction of Cr(VI) to Cr(III)^57^. As in the case of the photodegradation of MB, the composite was characterized by a better reduction efficiency of the chromium compound. The adsorption–desorption equilibrium was attained for 30 min in the dark. The results are presented in Fig. 7a.

The decrease in Cr(VI) concentration due to dark adsorption as well as during illumination was higher for the composite. The greatest decrease in Cr(VI) concentration in the case of NVO/rGO was observed in the first 15 min of illumination, and then the performance of the catalyst slowly stopped. As a result, after one hour of exposure, the photoreduction amounted to approximately 70% (in the case of NVO, only 20%). The modified material, namely, NVO/rGO, showed a greater ability to degrade the hazardous chromium form, which was due to the presence of rGO, which was responsible for more efficient separation and prolonged recombination time of electron–hole pairs. Moreover, as shown in Fig. 7b, the NVO/rGO composite displays good reusability and after four consecutive cycles, the degradation efficiency of Cr(VI) slightly decreases to 65%. The above experimental results demonstrate the great potential of the obtained NVO and NVO/rGO in the photodegradation of water pollutants. In contrast to the previous study^52^, it was shown that pristine NH_4_V_4_O_10_ can be efficiently used for the degradation of organic dyes. Moreover, the proposed combination of NH_4_V_4_O_10_ with rGO not only increases the photocatalytic degradation reaction rate and improves the cyclic stability of photocatalyst but also widens its application, allowing for the efficient photoreduction of toxic Cr(VI). In comparison to other vanadium-based photocatalysts reported in the literature^19,58–65^ (Tables S1 and S2), NVO and NVO/rGO present better or similar efficiency towards the photodegradation of MB and Cr(VI). Importantly, proposed photocatalysts can be activated under solar light and exhibit significant photocatalytic degradation of pollutants within the first 60 min of illumination. Such an efficient and fast process, together with facile synthesis (one-step reaction, low-cost precursors) and good cyclic stability of NVO and NVO/rGO make them promising materials for solar-driven water purification.

**Figure 8:**
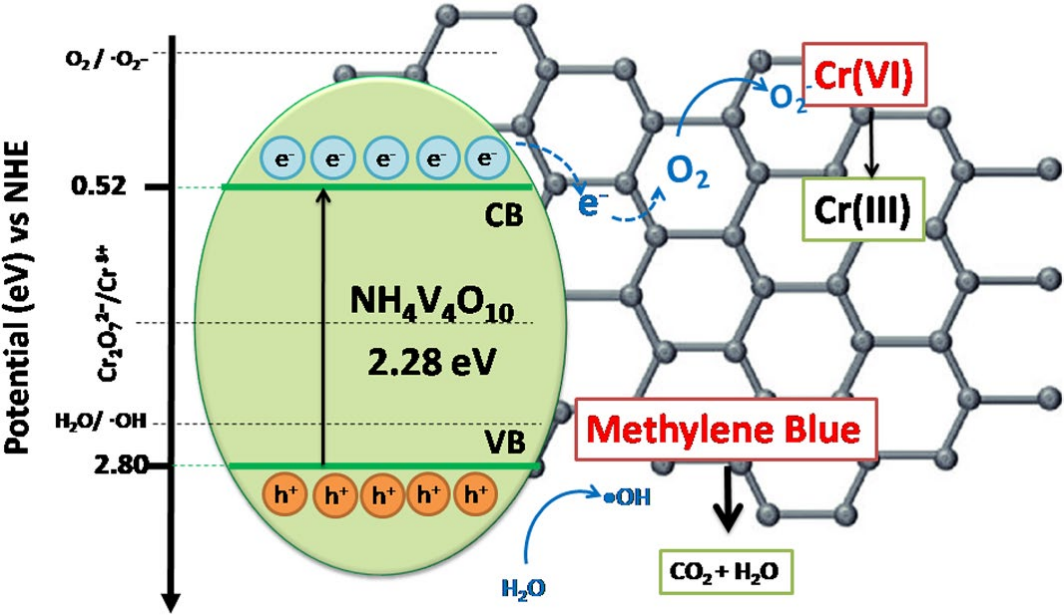
Schematic diagram of the band-energy levels of NVO/rGO with the possible photocatalytic mechanism.

Therefore, E_cb_ and E_vb_ were calculated to be 0.75 eV and 3.03 eV, respectively. On the basis of the obtained results, a possible mechanism for the photocatalytic performance of NVO and NVO/rGO has been proposed and is schematically presented in Fig. 8.

When NVO or NVO/rGO is exposed to solar illumination, NH_4_V_4_O_10_ becomes excited, and electron–hole pairs are generated. The photogenerated holes in the VB of NH_4_V_4_O_10_ can oxidize MB directly or react with water to produce (·OH), which are also able to indirectly oxidize organic species. At the same time, the E_cb_ of NH_4_V_4_O_10_ is lower than the reduction potential of O_2_ to ·O2- (-0.33 eV)^66^. However, in the case of the NVO/rGO composite, the photogenerated electrons can migrate from NH_4_V_4_O_10_ to rGO ^67,68^. It is known that the electrons at the reduced graphene oxide surface react readily with oxygen molecules and generate superoxide radical anions (·O2-) ^67^, which subsequently degrade the MB dye molecules. According to the results obtained from the active species trapping experiment, this process was the most dominant in NVO/rGO. On the other hand, the potential of the conduction band is more negative than the redox potential of the Cr_2_O_7_^2−^/Cr^3+^ couple (1.33 eV vs NHE), which indicates that NH_4_V_4_O_10_ can reduce Cr(VI) ions using their photoexcited electrons. The efficiency of Cr(VI) photoreduction for NVO equals only 20% after 60 min of illumination, while for NVO/rGO, it reaches 70%. The improved photocatalytic performance originates from the unique structure of the NVO/rGO composite, in particular the existence of rGO, which acts as an electron acceptor and inhibits the recombination of electrons and holes. In addition, the NVO/rGO composite is characterized by a narrower band gap and higher adsorption capacity of Cr(VI) than NVO.

## Conclusions

A one-pot hydrothermal method was successfully used to obtain NH_4_V_4_O_10_ (NVO) and its composite with rGO (NVO/rGO). The photocatalytic activity of synthesized NVO and NVO/rGO was evaluated towards methylene blue (MB) degradation and Cr(VI) to Cr(III) reduction under simulated solar light illumination. Excellent performance in MB photodegradation was obtained for both studied materials, which was attributed to the high content of V^4+^ surface species revealed by XPS studies, as well as the developed surface area of the photocatalysts. The positive impact of rGO in terms of the activity and stability of the photocatalyst was especially pronounced. Reactions carried out in the presence of specific scavengers revealed differences in the mechanism of photocatalyst action. In the case of NVO, ·OH and h + play a critical role in MB photodegradation, while for NVO/rGO, ·O2- radicals are the dominant active species responsible for dye degradation. Moreover, the proposed composite showed activity in the photoreduction of highly toxic Cr(VI) ions in an acidic environment and is therefore a promising photocatalyst for wastewater treatment containing both organic and inorganic contaminants. The hydrothermal synthesis parameters of NVO/rGO can cause different morphologies, particle sizes and crystallizations, resulting in different photocatalytic activities; therefore, such studies are planned to be carried out in our laboratory.

## Materials and methods

### Chemicals

Ammonium metavanadate (NH_4_VO_3,_ 99.0%), oxalic acid dihydrate (C_2_H_2_O_4_ × 2H_2_O, 97.0%), and methylene blue (MB > 98%) were obtained from Sigma‒Aldrich and used without further purification. Deionized water was used in all experiments (conductivity < 0,06 μS/cm). Graphene oxide (GO) employed in the composite synthesis was prepared using the modified Hummers method ^69^. Potassium dichromate (K_2_Cr_2_O_7_, ≥ 99.0%) and ammonium oxalate (AO, ≥ 99%) were purchased from Merck. Benzoquinone (BQ, > 98%) and *tert*-butyl alcohol (TBA, > 99.5%) were received from CheMondis.

### Synthesis of photocatalysts

The NH_4_V_4_O_10_/rGO (NVO/rGO) photocatalyst was prepared via a facile one-pot hydrothermal method. NH_4_VO_3_ and GO were used as precursors and mixed with a weight ratio of 10:1. In brief, 0.6 g of NH_4_VO_3_ and 0.6 g of oxalic acid were dissolved in 90 ml of deionized water. In the meantime, 60 mg of GO was dispersed in 30 ml of deionized water with ultrasonication (20 W, 30 min). Next, the prepared reagents were mixed together and sonicated for 15 min. Then, the as-obtained reaction mixture was transferred to a Teflon-lined stainless-steel autoclave (volume 1.8 L) for 8 h and 180 °C. Finally, the obtained product was washed with deionized water and dried at 40 °C under reduced pressure (0.01 bar). For comparison, bare NH_4_V_4_O_10_ (NVO) without GO was synthesized in an analogous procedure.

### Physicochemical characterization

The crystal structure and phase composition of the samples were examined using powder X-ray diffraction (XRD, BrukerD2 Phaser diffractometer) with Cu Kα radiation (λ = 1.5404 Å). Raman spectra were acquired using a confocal micro-Raman system (Horiba Jobin Yvon) with a 632.8 nm laser excitation wavelength. Fourier transform infrared (FTIR) analysis was carried out on a Perkin Elmer Frontier spectrophotometer in the range of 500–4000 cm^−1^. Measurements were made in transmittance mode, and the potassium bromide pellet method was used. The valence state of vanadium was analysed using X-ray photoelectron spectroscopy (XPS) and X-ray absorption spectroscopy (XPS). The high-resolution V 2p XPS spectra were collected on an Escalab 250Xi device (Thermo Fisher Scientific) equipped with a monochromatic AlKα source. Measurements were carried out at a 25 eV pass energy with a 0.1 eV energy step. The X-ray spot size was 250 µm. The calibration of the XPS spectrum was performed using the characteristic C1s peak at 284.6 eV. XAS analysis was performed at the ASTRA beamline at SOLARIS National Synchrotron Radiation Centre, Cracow, Poland. The V K-edge XANES of powder samples was obtained in transmission mode in the range of 5265 to 5550 eV. V_2_O_3_, VO_2_, and V_2_O_5_ were used as reference materials for the V(III), V(IV) and V(V) oxidation states. Thermogravimetric analysis (TGA) was conducted under air using a Netzsch STA 449 F1 at 10 °C/min from 40 °C to 600 °C. The morphology of the samples was investigated using scanning electron microscopy (SEM, ESEM Quanta Feg 250) and transition electron microscopy (TEM, FEI TECNAI G2 F20). N_2_ adsorption–desorption isotherms were measured on a NOVAtouch™ 2 surface analyser, and the Brunauer‒Emmett‒Teller (BET) method was used to calculate the specific surface area (relative pressure range p/p_0_: 0.1 to 0.3). The correlation coefficient of the linear regression was not less than 0.999. The pore size distribution and cumulative pore volume were evaluated using the BJH (Barrett, Joyner and Halenda) method from the desorption branch. The UV‒vis reflectance spectra of the selected materials were measured with a UV‒vis spectrophotometer (Lambda 35, Perkin-Elmer) equipped with a diffuse reflectance accessory. The spectra were registered in the range of 300–900 nm, with a scanning speed of 120 nm min^−1^. Band gap energy values were determined as the intercept of the x-axis of the plot of transformation of the Kubelka–Munk function. To determine the energy band gap (E_bg_) of the chosen powders, the Kubelka–Munk function was applied:

$$\mathrm{f}\left(\mathrm{KM}\right)=\frac{2\mathrm{R}}{{(1-\mathrm{R})}^{2}}$$

where R –reflectance. The bandgap was estimated by extrapolation of the linear region of (f(KM) hν)^n^ vs hν to y = 0. The power of n equals 2, assuming a direct band gap.

The PL spectra were measured using SCINCO FluoroMate FS-2 fluorescence spectrometer (excitation wavelength: 375 nm). Electrochemical measurements were accomplished using an Ivium Vertex potentiostat/galvanostat and a three-electrode cell with Pt mesh as the counter electrode and Ag/AgCl (3 M KCl) as the reference electrode. To perform these measurements, aqueous suspensions of NVO and NVO/rGO were drop-cast on glassy carbon disc electrodes. After drying with a hot stream of air, electrodes were tested in aqueous 0.2 M Na_2_SO_4_ solution. The EIS response was measured for 3 frequencies (500, 750, and 1000 Hz). The capacitance was estimated from the single points using the *C*_sc_ = -1/ω*Z*″ formula (ω – angular frequency, Z’’ – imagine part of impedance).

### Evaluation of photocatalytic activity

The photocatalytic behaviour was investigated by the oxidation of methylene blue and reduction of Cr(VI). The photocatalytic performance was evaluated under simulated solar light using a 300 W Xe lamp (a high-pressure 150 W xenon lamp, LOT – QuantumDesign GmbH equipped with the AM1.5G filter). The intensity of the incident light that reaches the surface of the investigated solution was equal to 100 mWcm^−2^ (measured using a Coherentâ FieldMate Laser Power Meter). In a typical test, 20 mg of catalyst was placed in a 50 mL aqueous pollutant solution. The concentration of MB and Cr(VI) was 1·10^–5^ M. Before irradiation, the suspension was vigorously stirred in the dark for 30 min to reach desorption-adsorption equilibrium. The change in MB and Cr(VI) concentration was monitored by its absorption at 665 nm and 351 nm, respectively, from the UV–Vis (Spektrofotometr UV5100) spectra of the solution, using distilled water as a reference. A total of 0.75 ml of suspension was collected and centrifuged before UV‒Vis measurement. In the case of Cr(VI) photoreduction, the process was conducted in acidified (pH = 3) solutions.

To study the reusability of the prepared photocatalysts, the cycle experiment was repeated 4 times for the photodegradation of methylene blue. After each photodegradation test, the catalyst was collected by centrifugation, dried under natural conditions and used for the next degradation experiment. Moreover, to indicate the role of hydroxyl radicals (·OH), (h +) holes and superoxide radicals (·O2-) in the process of MB degradation, experiments were performed in the presence of appropriate scavengers: t-butanol (TBA), ammonium oxalate (AO) and benzoquinone (BQ). The concentration of each scavenger was equal to 1 mM.

## Supplementary Information

Supplementary Information 1.

## Data Availability

All data generated or analysed during this study are included in this published article (and its Supplementary Information files).
