# Supplementary material for: New light on the photocatalytic performance of NH4V4O10 and its composite with rGO
Source: Sci Rep. 2023 Mar 9;13:3946. doi: 10.1038/s41598-023-31130-9 (PMC9998451; doi:10.1038/s41598-023-31130-9)
Supplement: Supplementary file 1 [file 41598_2023_31130_MOESM1_ESM.odt]

Supporting Information  

New light on the photocatalytic performance of NH4V4O10 and its composite with rGO

M. Nadolskaa, M. Szkodab, K. Trzcińskib, J. Ryla, A. Lewkowiczc, K. Sadowskad, , J. Smalc-Koziorowskae, M. Prześniak-Welenca*

aInstitute of Nanotechnology and Materials Engineering, and Advanced Materials Centre, Gdansk University of Technology, Narutowicza 11/12, 80-233 Gdansk, Poland,

bFaculty of Chemistry, Gdansk University of Technology, Narutowicza St. 11/12, 80-233 Gdansk, Poland

c Insitute of Experimental Physics, Faculty of Mathematics, Physics and Informatics, University of Gdańsk, Wita Stwosza 57, 80-308 Gdańsk, Poland

d Nalecz Institute of Biocybernetics and Biomedical Engineering, Polish Academy of Sciences, Ks. Trojdena 4, Warsaw, 02-109, Poland

e Institute of High Pressure Physics, Polish Academy of Sciences, Sokołowska 29/37, 01-142 Warsaw, Poland

* [marwelen@pg.edu.pl](mailto:marwelen@pg.edu.pl)

Figure S1. FTIR analysis of NVO and NVO/rGO

 

Figure S2. N2 adsorption-desorption isotherms with pore size distribution (inset) of NVO (left) and NVO/rGO (right).

Figure S3. Photoluminescence spectra of NVO and NVO/rGO

Figure S4. The cyclic voltammetry curves of NVO (a) and NVO/rGO (b), recorded in 0.2 M K2SO4, scan rate: 50 mV/s

Figure S5. Control tests for the photocatalytic degradation of MB: a) The adsorption of MB on the surface of NVO and NVO/rGO under dark conditions and b) photocatalytic removal of MB by rGO

Figure S6. Photocatalytic degradation of MB under simulated solar light-ln(C/C0) as a function of irradiation time representing the kinetics of the process.

Figure S7. Photocatalytic removal of Cr(VI) by rGO

Table 1 Comparison of MB degradation efficiency for different vanadium-based photocatalysts

| Photocatalyst | Efficiency (%) | Time (min) | Ref |
| --- | --- | --- | --- |
| BiVO4 | 60 | 120 | 58 |
| BiVO4/rGO | 90 | 120 | 58 |
| V2O5 | 34 | 90 | 59 |
| V2O5/rGO | 80 | 90 | 59 |
| AgVO3 | 42 | 150 | 60 |
| AgVO3/rGO | 91 | 150 | 60 |
| FeVO4 | 81 | 180 | 61 |
| KV3O8 | 92 | 60 | 19 |
| NH4V4O10 | 82 | 60 | Present work |
| NH4V4O10/rGO | 92 | 60 | Present work |

Table 1 Comparison of Cr(VI) photoreduction efficiency for different vanadium-based photocatalysts

| Photocatalyst | Efficiency (%) | Time (min) | Ref |
| --- | --- | --- | --- |
| BiVO4 | 61 | 330 | 62 |
| BiVO4/Bi2S3 | 100 | 150 | 62 |
| BiVO4 | 9 | 100 | 63 |
| La,Gd-BiVO4 | 47 | 100 | 63 |
| V2O5 | 3 | 90 | 64 |
| V2O5 /g-C3N4 | 71 | 90 | 64 |
| AgVO3 | 10 | 25 | 65 |
| AgVO3/ZnIn2S4 | 90 | 25 | 65 |
| NH4V4O10 | 20 | 60 | Present work |
| NH4V4O10/rGO | 71 | 60 | Present work |
